# Comparing the effectiveness of full-vacuum and half-vacuum drainage in reducing seroma after modified radical mastectomy: a meta-analysis

**DOI:** 10.1308/rcsann.2024.0034

**Published:** 2024-05-09

**Authors:** S Oyewale, A Ariwoola

**Affiliations:** ^1^ University of Ilorin Teaching Hospital, Nigeria; ^2^ Rutgers School of Public Health, New Brunswick, NJ, US

**Keywords:** mastectomy, drain, effectiveness, breast cancer, seroma

## Abstract

**Introduction:**

Owing to limited outpatient support for drain management, delayed discharge after mastectomy is more prevalent in developing countries. Utilising half-vacuum (HV) suction drains for routine drainage after mastectomy could lead to a reduced incidence of seroma and a shorter hospital stay. This systematic review and meta-analysis compared the outcomes of HV against full-vacuum (FV) suction drains in patients who underwent modified radical mastectomy for breast cancer.

**Methods:**

Differences between the two groups in length of hospital stay, total volume of drain effluent and incidence of seroma were assessed. RevMan 5.4 was used to calculate the odds ratio (OR) and relative risk (RR) for dichotomous data, and the mean difference (MD) for continuous data.

**Results:**

Nine randomised controlled trials were included in this review. Using HV drains reduced both the mean length of hospital stay (MD: −2.30 days, 95% confidence interval [CI]: −4.10 to −0.49 days, I^2^=97%) and the mean total volume of effluent (MD: −132.61ml, 95% CI: −207.32ml to −57.91ml, I^2^=88%) compared with FV drains. However, there was no statistically significant difference in incidence of seroma between the two groups (RR: 0.67, 95% CI: 0.30 to 1.46, I^2^=65%). Likewise, there was no difference in rate of seroma occurrence on sensitivity analysis (OR: 1.29, 95% CI: 0.72 to 2.33, I^2^=74%).

**Conclusions:**

There was no difference in the incidence of seroma between HV and FV suction drainage. Conversely, a reduction in the length of hospital stay and the total volume of drain effluent was observed for mastectomy patients with a HV drain.

## Introduction

Seroma and haematoma are among the most significant early complications associated with mastectomy.^1^ In order to reduce the morbidity associated with seroma and promote the apposition of flaps, the use of a drain in the immediate postoperative period has been in vogue since 1947,^2^ when the first drain was reportedly used after mastectomy. A multitude of techniques and methods have been employed to prevent or reduce the incidence of post-mastectomy seroma with varying degrees of success.^1,3–5^

Use of a full-vacuum (FV) suction drain may prevent the closure of the lymphatic vessels with drainage of a high volume of effluent, potentially resulting in a longer hospital stay.^6^ Routine use of a FV drain might therefore be responsible for a reduced availability of bed spaces for the treatment of other surgical pathologies, especially in developing countries. Moreover, Bozzetti *et al* found that prolonged serous discharge after mastectomy can lead to persistent loss of albumin with consequent hypoalbuminaemia.^7^ This could harm wound healing, and may delay the commencement of adjuvant chemotherapy and radiotherapy.

Early discharge of patients after mastectomy has led to significant cost savings and psychological benefits without compromising the quality of care.^8^ In low and middle-income countries, where there are very few community nurses for home visitations, routine use of FV drainage might be responsible for a longer hospital stays as discharging patients home with a drain is challenging owing to the low literacy rate. For this reason, patients who have mastectomies in developing countries could have a prolonged hospital stay of up to ten days after surgery,^9^ which would allow the volume of drain effluent to reduce considerably before drain removal.

Given that the use of FV suction drains has been associated with seroma formation,^10–12^ routine use of half-vacuum (HV) drainage after mastectomy may reduce the overall volume of drain effluent and therefore allow early discharge. In addition, there has been no meta-analysis comparing the effectiveness of HV drains with FV drains. The aim of this study was to compare the complication profiles of these two types of drain after modified radical mastectomy in breast cancer patients. Furthermore, the evidence synthesised with regard to the effectiveness of HV drains might encourage their routine use in patients who have modified radical mastectomy for breast cancer.

## Methods

The research question for this review was: After modified radical mastectomy for breast cancer, is there any difference in the incidence of seroma formation between patients with a HV and those with a FV suction drain? This review was registered in the PROSPERO database (CRD42023428415).

The primary outcome measure was the incidence of seroma. The secondary outcome measures included the total volume of drain effluent and the length of hospital stay. The comparator group comprised patients with a FV suction drain (700g/m^2^ or 100mmHg) while the intervention group comprised those with a HV drain (350g/m^2^ or 50mmHg).

The PubMed^®^, Google Scholar™ and Directory of Open Access Journals databases were searched using the search terms shown in Tables 1 and 2. The literature search started on 15 June 2023 and was completed on 20 July 2023.

**Table 1:** Search strategy for PubMed^®^ database

	Search terms
#1	full vacuum drain OR high pressure drain
#2	half vacuum drain OR half pressure drain
#3	mastectomy with axillary dissection OR modified radical mastectomy
#4	length of hospital stay OR duration of hospital stay
#5	seroma
#6	volume of drain effluent
#7	((((full vacuum drain OR high pressure drain) AND (half vacuum drain OR half pressure drain)) OR (mastectomy with axillary dissection OR modified radical mastectomy)) AND (length of hospital stay OR duration of hospital stay) AND (seroma OR volume of drain effluent))

**Table 2:** Search strategy for Google Scholar™ and Directory of Open Access Journals (DOAJ) databases

	Search terms
Google Scholar™	full pressure drain AND half pressure drain AND modified radical mastectomy AND seroma AND volume of drain
DOAJ	full vacuum drain AND half vacuum drain AND mastectomy AND seroma

**Table 3:** Summary of the included studies

Study	Country	Type of study	Number of patients	Mean patient age	Level of axillary dissection	Drain pressure	Indication for drain removal
Athar, 2020^18^	India	RCT	FV: 42 HV: 34	Not available	Level II/III	FV: 700g/m^2^ HV: 350g/m^2^	Not available
Chintamani, 2005^6^	India	RCT	FV: 50 HV: 35	FV: 49.5 years HV: 46 years	Level III	FV: 700g/m^2^ HV: 350g/m^2^	<30ml/day
Ganiger, 2021^19^	India	RCT	FV: 20 HV: 20	FV: 47.1 years HV: 46.3 years	Not available	FV: 700g/m^2^ HV: 350g/m^2^	Not available
Imam, 2020^20^	India	RCT	FV: 40 HV: 40	Not available	Level III	FV: 100mmHg HV: 50mmHg	<10ml/day
Ivan, 2022^21^	Indonesia	RCT	FV: 13 HV: 13	FV: 49.5 years HV: 52.5 years	Not available	FV: 700g/m^2^ HV: 350g/m^2^	Not available
Kakde, 2022^22^	India	RCT	FV: 20 HV: 35	FV: 58.6 years HV: 51.6 years	Level III	FV: 700g/m^2^ HV: 350g/m^2^	<30ml/day
Lal, 2017^23^	India	RCT	FV: 25 HV: 25	Not available	Level III	FV: 100mmHg HV: 50mmHg	<10ml/day
Mohan, 2023^24^	India	RCT	FV: 50 HV: 50	FV: 51.4 years HV: 51.4 years	Level III	FV: 700g/m^2^ HV: 350g/m^2^	<30ml/day
Singh, 2019^25^	Indonesia	RCT	FV: 15 HV: 15	Not available	Not available	FV: 700g/m^2^ HV: 350g/m^2^	Not available
FV = full vacuum; HV = half vacuum; RCT = randomised controlled trial							

### Study selection and data collection process

Both authors (SO and AA) were involved in article screening. This was carried out independently. Rayyan software was employed for screening and selection.^13^ Duplicate records were deleted and the full text of the remaining articles was assessed. The inclusion criteria (see below) were used to settle any disagreements between the authors. The two authors independently extracted the following data from the papers included in the review:

- Basic information – first author, year of publication and study location
- Demographic information – number of study participants, mean patient age and drain removal protocol
- Outcomes – seroma incidence, length of hospital stay and volume of drain effluent

The collected data were subsequently imported into an Excel^®^ spreadsheet (Microsoft, Redmond, WA, US). The extracted data were then compared jointly by the authors for consensus and accuracy.

### Inclusion and exclusion criteria

The inclusion criteria comprised randomised controlled trials comparing HV with FV suction drains in modified radical mastectomy published in English since 1990. Studies comparing axillary clearance, breast-conserving surgery and mastectomy performed in conjunction with breast reconstruction were excluded, as were those using continuous suction drainage.

### Risk of bias in individual studies

The risk of bias was evaluated with the revised Cochrane RoB 2 tool.^14^ This was undertaken by the two authors (SO and AA) independently. The elements assessed included bias arising from the randomisation process, bias due to deviation from the intended intervention, bias due to missing outcomes data, bias in the measurement of the outcomes and bias in selection of the reported results. The data were subsequently compared and a consensus was reached whenever there was a difference in opinion.

The data were tabulated and incorporated into the robvis (Risk-Of-Bias VISualization) tool to provide a visual summary of the risk of bias for the included studies.^15^ It was not appropriate to assess publication bias with a funnel plot/Egger's test as fewer than ten studies were incorporated in the review, meaning that the test might result in a type II error owing to its power being low.^16^

### Summary measures

The odds ratio (OR) and relative risk (RR) were calculated for dichotomous data while the mean difference (MD) was used for continuous data.

### Synthesis of results

RevMan 5.4 (Nordic Cochrane Centre, Copenhagen, Denmark) was employed to analyse the data. The I^2^ statistic was calculated to assess the heterogeneity of the collected data. The random-effects model was used for analysis. Heterogeneity was explored based on the drain removal protocol. Whenever the mean was not stated in a study, it was calculated from the median and range using the formula developed by Hozo *et al*.^17^

### Additional analysis

A sensitivity analysis relating to the data on seroma incidence was undertaken by eliminating studies with moderate to high risk of bias.

## Results

**Figure 1:**
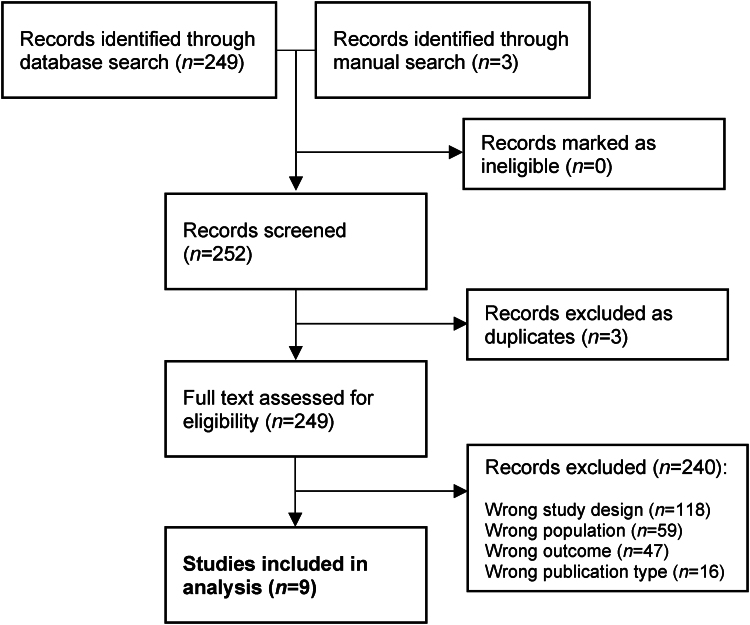
Flowchart of study selection

The initial literature search identified 252 articles. After excluding 3 duplicate records, full-text review was conducted for 249 studies (Figure 1). Of these, 9 studies comprising 542 patients were included in our review (Table 3).^6,18–25^ All the studies were randomised controlled trials based in Asia.

### Risk of bias

**Figure 2:**
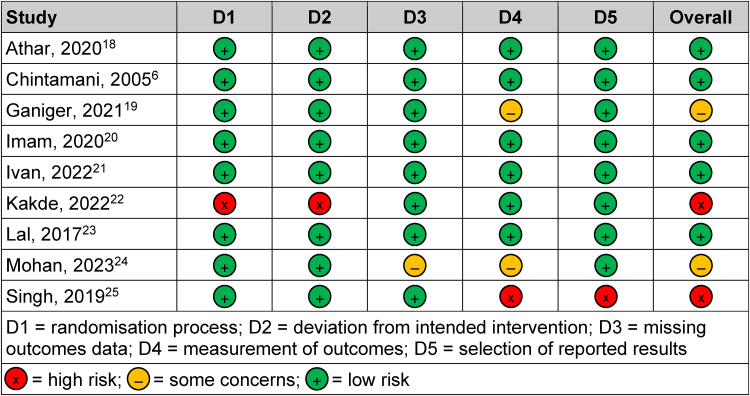
Traffic light plot for the risk of bias of the included studies

**Figure 3:**
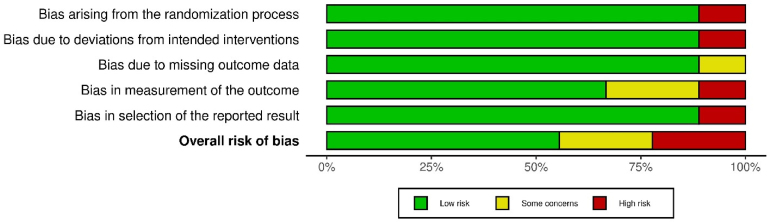
Summary plot for the risk of bias of the nine included studies^6,18–25^

The RoB 2 tool was employed to assess the studies' risk of bias.^14^ Two of the nine studies were found to have a high risk of bias (Figure 2).^22,25^ In the study by Singh *et al*, there were biases in the measurement of the outcomes and in the selection of the reported results.^25^ In addition, the study by Kakde *et al* had biases associated with the randomisation process and deviation from the intended intervention.^22^ The summary plot gives a general picture of the bias risk of the included studies (Figure 3).

### Length of hospital stay

**Figure 4:**
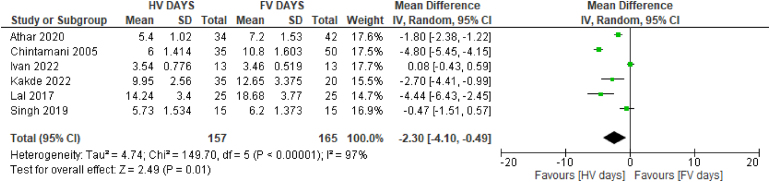
Forest plot comparing mean length of hospital stay across six studies^6,18,21–23,25^

The analysis for the length of hospital stay following modified radical mastectomy was derived from six studies.^6,18,21–23,25^ There was a significant reduction in the mean length of stay when HV suction drainage was used (MD: −2.30 days, 95% confidence interval [CI]: −4.10 to −0.49 days, I^2^=97%) (Figure 4).

### Volume of drain effluent

**Figure 5:**
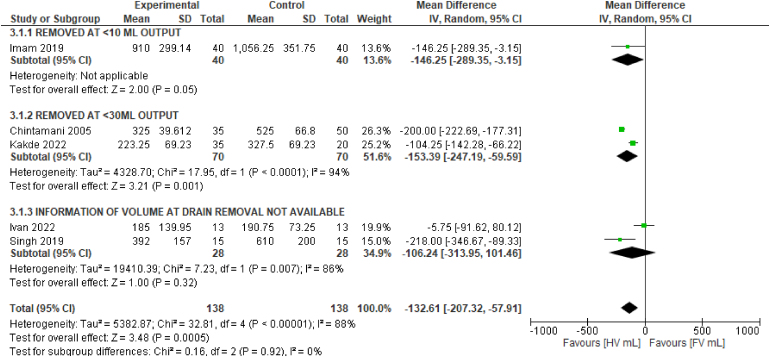
Forest plot comparing mean total volume of drain effluent across five studies,^6,20–22,25^ with subgroup differences based on drain removal protocol

The data for total volume of drain effluent were derived from five studies.^6,20–22,25^ Use of HV drainage reduced the overall mean total volume of effluent (MD: −132.61ml, 95% CI: −207.32ml to −57.91ml, I^2^=88%). The drains were removed according to different protocols in different studies. Statistically significant reductions in volume of effluent were noted both in studies where the drain was removed at an output of <10ml/day and in those where it was removed at an output of <30ml/day. The greatest reduction was observed in the latter (MD: −153.39ml, 95% CI: −247.19ml to −59.59ml, I^2^=94%). The test for subgroup differences was not statistically significant (*p*=0.92), indicating that the drain removal protocol did not modify the effects of the drains on the total volume of effluent (Figure 5).

### Incidence of seroma

**Figure 6:**
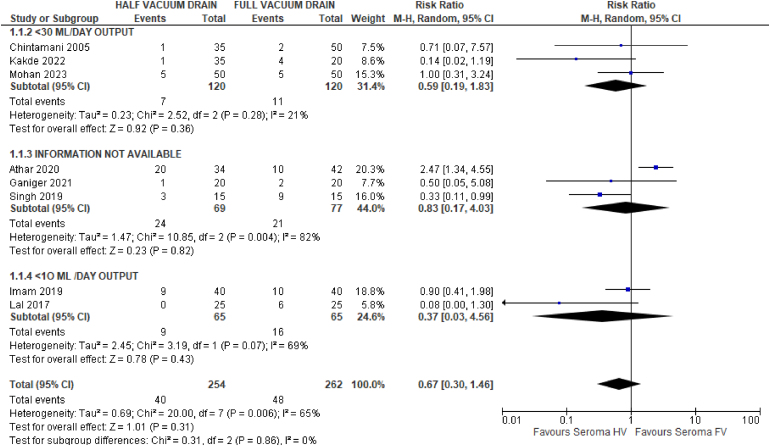
Forest plot comparing incidence of seroma across eight studies,^6,18–20,22–25^ with subgroup differences based on drain removal protocol

The data for seroma incidence were derived from eight studies.^6,18–20,22–25^ The 95% CI crossed the line of no effect, indicating that there was no difference between HV and FV drainage in terms of seroma formation (RR: 0.67, 95% CI: 0.30 to 1.46, I^2^=65%) (Figure 6).

**Figure 7:**
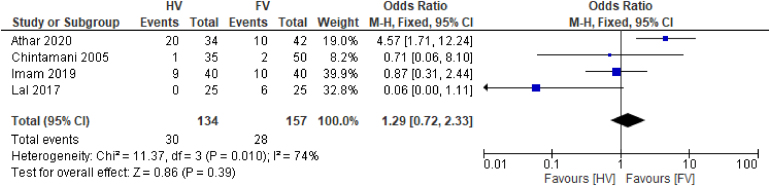
Forest plot of sensitivity analysis of incidence of seroma across four studies^6,18,20,23^

Sensitivity analysis did not demonstrate any difference in incidence of seroma between HV and FV drains after removal of studies with moderate or high risk of bias (OR: 1.29, 95% CI: 0.72 to 2.33, I^2^=74%) (Figure 7).^6,18,20,23^

## Discussion

Many patients prefer an early discharge owing to difficulty sleeping because of noise in the hospital and dislike of the hospital environment.^26^ In addition, a prolonged hospital stay can significantly reduce patient satisfaction^27^ as well as exposing patients to potential hospital-acquired infections and therefore increasing healthcare costs.^28^

Since the use of drains has been associated with patient discomfort,^29^ early removal of HV suction drains could lead to improved patient comfort and satisfaction. For this reason, in developing countries, routine use of HV drainage after mastectomy could lead to a shorter hospital stay, and allow prompt commencement of chemotherapy and radiotherapy. This might also translate to a reduction in the overall treatment cost, which could be significant in low and middle-income countries, where the majority of patients do not have health insurance. Furthermore, there is a possibility that use of a HV drain after mastectomy might lead to a shorter hospital stay and a reduced incidence of seroma.

The lower volume of effluent with HV drainage suggests that the FV drain may cause opening of the lymphatic channels.^6^ Consequently, HV drainage is associated with a reduced volume of effluent and might lead to a better quality of life.^30^

There are various risk factors responsible for seroma formation after mastectomy. These include the volume of drainage in the first 72 hours, and patient age and weight.^31^ The use of electrocautery has also been associated with a higher incidence of seroma^32^ owing to the effect of heat on subcutaneous fat^33^ and the partial sealing of the lymphatic vessels.^34^ There is no difference in the therapeutic effects of HV and FV suction drains in reducing seroma formation.

### Study limitations

There are certain limitations to this review that should be acknowledged. The included studies showed variation in their patient characteristics, sample sizes, flap-raising techniques and drain management protocols. In addition, the studies were based on patients exclusively in Asian countries and the sample sizes in some of the included studies were relatively small. These factors may limit the generalisability of the findings in this review. Furthermore, it was not possible to assess publication bias because there were fewer than ten studies included in the review.

The use of a HV drain after mastectomy in developing countries could lead to shorter hospital stays and prompt commencement of adjuvant treatment for breast cancer, with a subsequent reduction in overall treatment costs. Future well-designed randomised controlled trials comparing HV and FV drainage with larger sample sizes and standardised protocols for drain removal are needed to provide more conclusive evidence with regard to the effects of different types of drain on seroma formation in patients after mastectomy.

## Conclusions

This meta-analysis suggests that use of a HV suction drain results in a shorter hospital stay and reduced total volume of effluent than use of FV drainage. Even though the observed seroma incidence was higher in the HV cohort than in the FV cohort, this was not statistically significant, indicating that the use of FV drains after modified radical mastectomy for breast cancer may not be superior in preventing seroma compared with HV drainage. Moreover, a FV drain may even reduce the patient’s chances of early discharge.
